# A Tissue-Specific Scaffold for Tissue Engineering-Based Ureteral Reconstruction

**DOI:** 10.1371/journal.pone.0120244

**Published:** 2015-03-16

**Authors:** Yongde Xu, Weijun Fu, Zhongxin Wang, Gang Li, Xu Zhang

**Affiliations:** 1 Department of Urology, Hainan Branch of Chinese People’s Liberation Army General Hospital, Sanya, China; 2 Department of Urology, Chinese People`s Liberation Army General Hospital, Beijing, China; 3 Institute of Organ Transplantation of PLA, 309th Hospital of PLA, Beijing, China; Northwestern University Feinberg School of Medicine, UNITED STATES

## Abstract

Terminally differentiated somatic cells can rapidly change phenotypes when they are isolated from their native tissue and cultured *in vitro*. This problem may become a barrier to tissue engineering-based organ reconstruction, which utilizes somatic cells. The present study was designed to validate the feasibility of maintaining the urothelial cell phenotype in a tissue-specific ureteral scaffold. The tissue-specific scaffold was fabricated by blending poly (L-lactic acid) (PLLA) and ureteral extracellular matrix (UECM) using electrostatic spinning technology. PLLA was used to enhance the mechanical properties, and UECM was used to mimic the natural components of the ureter. Primary urothelial cells (UCs), derived from ureteral mucosa, were seeded onto the tissue-specific scaffold to assess cell adhesion, proliferation and phenotypes at designated time points. The results showed that UCs in the tissue-specific scaffold exhibited better proliferation compared to cells in pure PLLA or a PLLA-small intestinal submucosa (PLLA-SIS) scaffold (*p*<0.05). At different time points, the expression of a UC-specific marker (UroplakinⅢ) in the tissue-specific scaffold was significantly higher than its expression in pure PLLA or a PLLA-SIS scaffold (*p*<0.05). Therefore, the tissue-specific scaffold appears to be an ideal substrate for promoting UC survival and phenotype maintenance.

## Introduction

The permeability barrier of the urinary tract is mainly attributed to the asymmetrical unit membrane (AUM), which is located on the luminal surface of urothelial cells (UCs). The AUM, which consists of uroplakin proteins (UPs) [1,2], exhibits low permeability to water, urea, NH_3_, H^+^, and small non-electrolytes. The normal permeability properties of UCs are necessary for urinary tract function and metabolic homeostasis. Therefore, it is crucial to maintain the phenotypic expression of UPs when urinary tract reconstruction is conducted using tissue-engineering methods. Currently, the isolation and *in vitro* expansion of primary UCs has become routine [3,4]. Unfortunately, it is difficult to form a differentiated, functional urothelium from adherent cultured UCs *in vitro* because terminally differentiated somatic cells rapidly lose some of their phenotypic traits after being removed from their native tissue [5–7].

Recently, the use of tissue-specific extracellular matrix (ECM) to improve cell proliferation and induce stem cell differentiation has drawn considerable attention [8–11]. The structural and functional components of the ECM allow adjacent cells to communicate with each other and with the extracellular environment. This communication plays a fundamental role in cell adhesion, spreading, proliferation, organ development and damage repair [12–14]. However, the composition of the ECM is complex and has not been fully characterized. As a result, it is difficult to synthesize artificial ECM in the laboratory. Poly (L-lactic acid) (PLLA) has been used widely as a biomaterial because of its good mechanical properties, biodegradability, biocompatibility and low toxicity [15,16]. However, PLLA does not have a favorable surface for cell attachment and proliferation because of its hydrophobicity as well as its lack of specific signals that can be recognized by cells [17].

In the present study, we fabricated a tissue-specific scaffold by blending ureteral ECM with PLLA using an electrostatic spinning method. We hypothesized that the ureter-specific electrospun nanofiber scaffold would maintain the specific phenotype of UCs when they were cultured *in vitro*. The aim of the study was to investigate the feasibility of using this tissue-specific scaffold for tissue engineering-based ureteral reconstruction.

## Materials and Methods

### Ethics statement

This study was approved by the institutional review board of the Center for People's Liberation Army General Hospital, Military Postgraduate Medical College. All participants provide their written consent to participate in this study.

### Tissue harvest and primary UCs isolation

8 patients (age: 30–62 years; mean age: 45.3 ± 11.7 years) with localized renal cell cancer (stage T2) underwent radical nephrectomy and were included in this study. Samples (about 2cm) for primary isolation of UCs were obtained from the end of the excised ureter. The tissue samples were kept at 4°C until further processing. The excess fat and connective tissue were removed from the samples under a stereo-microscope. The tissue samples were then microdissected into pieces and treated with 0.25% trypsin (Invitrogen, Auckland, CA) in PBS containing 0.02% EDTA (Sigma, USA) for 30 mins at 37°C. The enzyme activity was stopped by adding Dulbecco’s modified Eagle’s medium (DMEM) plus 10% fetal bovine serum (FBS, Invitrogen, USA). The cell suspension was filtered through 100 micrometer nylon mesh (Becton Dickinson, Durham, USA) and centrifuged at 220 relative centrifugal forces (RCF) for 5 mins. The cells were resuspended and cultured in complete defined keratinocyte serum-free medium (KSFM) (Invitrogen Ltd, Paisley, UK) supplemented with bovine pituitary extract and human recombinant epidermal growth factor (Sigma, USA) at the concentrations recommended by the manufacturer. The cells were incubated at 37°C in a humidified atmosphere with 5% CO_2_. The medium was changed every 2 days. In this study, each group contained all eight patients and UCs from each patient were separately analyzed in each experiments. Finally, the mean values of each group were shown in the Table 1.

**Table 1:** Designated time points and sample values.

Items	Time point /days after seeding	Sample value/ each time point per group
MTT	1, 3, 5, 7 d	n = 3
Live/Dead assay	2 d	n = 6
HE, IF	14 d	n = 6
WB	5, 14 d	n = 6

### Preparation of decellularized ureteral ECM and SIS powder

After UCs isolation via trypsin digestion, trypsin activity was quenched by adding DMEM containing 10% fetal bovine serum (FBS). The cell suspension was filtered through cell strainer (200 mesh), centrifuged for 5 min at 1,500 rpm and rinsed with phosphate buffer solution (PBS) for three times. Then, the debris left over was further decellularized by exposure to a digestion solution with continuous stirring. The digestion solution contained 1% Triton X-100 (Bio-Rad, USA) and 0.02% EDTA (Sigma, USA) in PBS. The decellularized procedure continued 72 h, and the digestion solution was changed every 24 hrs. SIS-ECM was fabricated as previously described [18]. Briefly, porcine intestines were rinsed with water and the mesenteric tissues were removed. The tunica serosa, tunica muscularis externa, and the luminal portion of the tunica mucosa were removed. Then, the tunica submucosa and the basilar layer of the tunica mucosa remained. The remained material was further decellularized using the same digestion solution and following the same procedure. The decellularized ureteral ECM or SIS was then rinsed in PBS for 2 days and in deionized water for 1 day. After decanting the liquid, the samples were frozen for at least 1 day at -70°C. The frozen samples were lyophilized, powderized using a micro-grinder and dissolved in 2 M urea. Undissolved ECM was removed by centrifugation at 3300 RCF for 20 mins. The supernatant was then filtered through a 40μm filter to further clarify. Finally, the solution was dialyzed against distilled water, frozen at -70°C and lyophilized again.

### Histological analysis and immunostaining

Fresh ureter specimens and decellularized ureteral ECM were used for immunofluorescence (IF) and histological analysis. For IF, the tissues were fixed with 2% formaldehyde and 0.002% picric acid in 0.1 M PBS for 4 hrs, followed by immersion in 30% sucrose in PBS overnight at 4°C. The fixed tissue specimens were then embedded in an optimal cutting temperature compound (Sakura Finetek, Torrance, CA) at -20°C, cut into 5μm thick sections, and mounted on glass slides. The slides were washed twice in PBS and then incubated with 5% goat serum in 0.3% Triton X-100 for 30mins at room temperature. The sections were incubated with primary antibodies overnight at 4°C, followed by 1 h incubation in a 1:500 dilution of secondary antibody conjugated with either Alexa Flour 488 (Invitrogen) or Texas Red. The primary antibodies were rabbit anti-cytokeratin AE1/AE3 (CK AE1/AE3, 1:50, Santa Cruz Biotechnology) and anti-uroplakinIII (UPIII, 1:50, Santa Cruz Biotechnology). Nuclear staining was performed with 4', 6-diamidino-2-phenylindole (DAPI). For histological analysis, the specimens were fixed in 4% paraformaldehyde, embedded in paraffin, cut into 5μm thick sections, and mounted on positively charged slides. Hematoxylin and eosin (H&E) as well as Masson’s trichrome staining were performed using standard histological techniques.

### Scaffold fabrication by electrostatic spinning

Base on our previous study[19], electrospun nanofiber meshes were fabricated using a blend of PLLA (mean molecular weight 1.5 × 10^5^, ShanDong Medical Co., China) and UECM/SIS with a ratio of 1:1 in weight. Both PLLA and UECM/SIS were dissolved in hexafluoroisopropanol (HFIP) at a total concentration of 6% (wt/vol). The solution was delivered by a programmable syringe pump to an electrically charged needle at a flow rate of 1.0 ml/h. The electro-spinning process was performed at a high voltage of 18–24 kv between the needle tip and the grounded surface. Fibers were ejected toward a grounded tinfoil plate at a distance of 10cm. The resulting nanofibrous membranes were stored for 48 hrs in a vacuum oven to remove residual solvent. Finally, the membranes were dried in a vacuum oven at 37°C for 3 weeks and sterilized with ethylene oxide prior to scaffold seeding.

### Scaffold seeding

In the present study, primary cultured (passage 0) UCs were used for experiments. After 8 days of incubation, UCs were resuspended and used for subsequent operations. The scaffolds were seeded as described in our previous work [19]. Briefly, a sterilized nanofibrous membrane was curved into a cylinder and inserted into a centrifuge tube. Primary UCs were adjusted to a concentration of 1 × 10^7^ cells/ml with KSFM. The cell suspension was then added to the centrifuge tube and centrifuged at 1856 RCF for 10 mins. Pure PLLA scaffolds and PLLA-SIS scaffolds were used as controls. The seeded scaffolds were harvested aseptically, removed from the tinfoil and incubated in 10cm culture dishes containing 15ml media by which the seeded scaffolds were suspended in at 37°C in a humidified atmosphere with 5% CO_2_. Scaffolds from each group were randomly selected for performing an MTT assay and other analyses at designated time points.

### Cells survival and proliferation analysis

After 48 h of incubation, seeded scaffolds were cut into squares with an area of 1cm^2^ and stained using the Live/Dead Viability/Cytotoxicity assay kit. Briefly, the cells were incubated for 30 mins with 2 mmol/L calcein-AM and 4 mmol/L ethidium homodimer-1 in a 37°C, 5% CO_2_ incubator. The scaffolds were rinsed gently with PBS and visualized immediately using a fluorescence microscope.

NAD(P)H-dependent cellular oxidoreductase enzymes are capable of reducing the tetrazolium dye MTT [3-(4,5-dimethylthiazol-2-yl)-2,5-diphenyltetrazolium bromide] to its insoluble formazan, which has a purple color. The MTT assay is a colorimetric assay for assessing cell viability, under defined conditions, it reflect the number of viable cells present.

To detect the proliferation of the UCs, 12 seeded scaffolds with an area of 0.16 cm^2^ (0.4cm×0.4cm) from each group were selected for MTT assay at day 1, 3, 5 and 7 (n = 3, per time point). The cells were seeded at a density of 4 × 10^4^ cells/cm^2^. The seeded scaffolds were incubated for 4 h in a 6-well containing 5ml media at 37°C and then transferred to a 96-well plate. Then, 20μl of MTT and 200μl of KSFM were added to each well. The 96-well plate was incubated for 4 hrs in a humidified atmosphere at 37℃ with 5% CO_2_. Then, the solution was gently aspirated from the wells. 150μl of DMSO (dimethyl sulfoxide) was added to dissolve the formazan crystals created by viable cells. Aliquots of the dissolved formazan solution were transferred to blank wells for spectrophotometric analysis at 570nm. Cell-free scaffolds (n = 3, per time point) in 150μl of DMSO served as blank control. Absorbance values of known cell numbers (2500, 5000, 10000, 20000 and 50000 per scaffold) were measured with an enzyme-labeling measuring instrument. Standard curves of the MTT value versus cell number (r^2^ ≥ 0.99≥0.95; cell number per scaffold = 0.16 × × cell concentration, surface area of each scaffold = 0.16cm^2^) were made direct to the corresponding scaffold. The number of cells for each group was calculated using the equation generated by the specific standard curve for each scaffold.

### Western Blot

After 5d and 14d of incubation, protein samples were prepared by homogenizing the seeded scaffolds (1cm × 2cm per scaffold) in RIPA lysis buffer. Equal amounts of cell lysate containing 20μg protein were electrophoresed by 10% sodium dodecyl sulfate polyacrylamide gel electrophoresis and then transferred to a polyvinylidene fluoride membrane (Millipore Corp, Bedford, MA, USA). The membrane was blocked with 5% skim milk for 1 h at room temperature and incubated overnight at 4°C with primary antibodies against GAPDH (Santa Cruz Biotechnology; CA, USA. 1:1000), CK AE1/AE3 (1:100, Santa Cruz Biotechnology), and UPIII (1:100, Santa Cruz Biotechnology).

### Image and statistical analysis

Designated time points and sample values are shown in Table 1. All histological results were evaluated blindly by two experienced pathologists. For image analysis, 5 randomly selected fields of a scaffold in each group (the PLLA-ureteral extracellular matrix (PLLA-UECM) group, the PLLA-SIS group and the PLLA group) were recorded and semi-quantitatively analyzed with Image Pro Plus 6.0 (IPP) imaging software. The percentage of live seeded cells in each group was calculated using the number of green cells compared to the total number of cells (red plus green). Integral optical density (IOD) of the pictures was analyzed by IPP 6.0 software to semi-quantitatively evaluate the expression of CK AE1/AE3 and UPIII. IOD of the WB image was also analyzed by IPP 6.0 for semi-quantitatively evaluation. All numerical data are expressed as the mean ± SEM (the standard error of the mean). The different groups tested were compared using a one-way analysis of variance followed by Tukey HSD post hoc comparisons, and *p*<0.05 was considered significant.

## Results

### Histological analysis of the decellularized matrices

**Fig 1:**
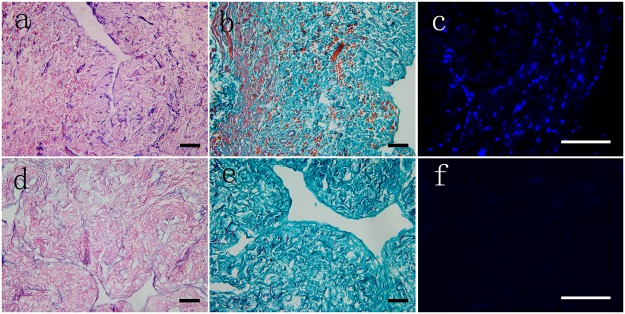
Histological analysis of fresh ureter tissue and its corresponding decellularized ECM via H&E, Masson’s trichrome and DAPI staining. Cellular debris was obvious in the fresh tissue cross-sections (a, b) but had disappeared in the decellularized ECM (d, e). Intact and well-organized nuclei could be observed in the fresh tissue (c), whereas none could be seen in the decellularized ECM (f). Inset scale bar = 300 μm.

Cross-sections were histologically analyzed to evaluate the efficiency of decellularization. In fresh samples, abundant cell component were visualized by H&E (Fig. 1a), Masson’s trichrome (Fig. 1b) and DAPI (Fig. 1c) staining. After decellularization, cellular remnants were rarely observed (Fig. 1d–1e). No intact nuclei could be seen within the decellularized matrices by DAPI staining (Fig. 1f).

### Viability of the seeded UCs

**Fig 2:**
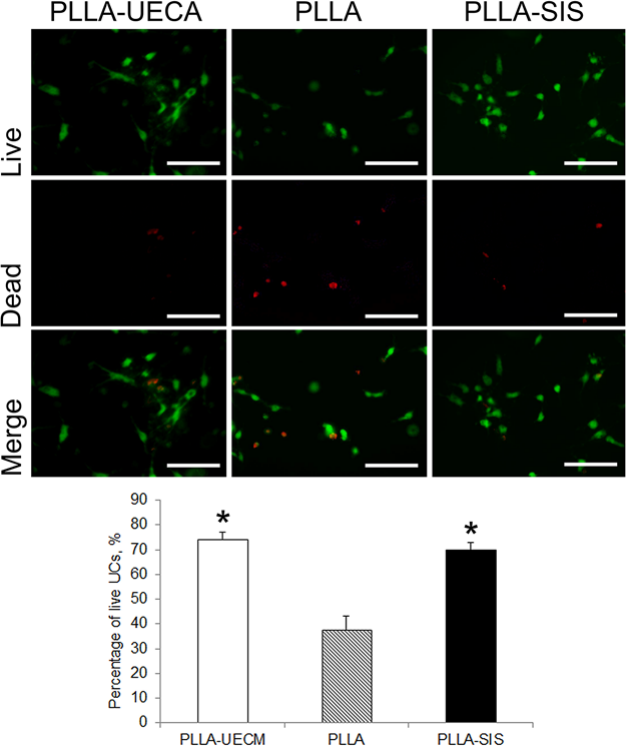
Live/dead staining was used to analyze the fates of the seeded cells. The number of surviving cells on the PLLA-UECM and PLLA-SIS scaffolds were significantly higher than that on the pure PLLA scaffold (*p*<0.05). Inset scale bar = 100 μm. *: *p*<0.05 compared with the PLLA group.

The percentage of live cells was calculated as the ratio of live cells (green) to the total number of cells (red plus green). The data of each group were calculated from 30 randomly chosen fields of 6 scaffolds in each group. There were more live cells in the PLLA-UECM scaffold and the PLLA-SIS scaffold compared to the pure PLLA scaffold. Two days after seeding, live/dead staining showed there were more live cells in the PLLA-UECM scaffold and the PLLA-SIS scaffold compared to the pure PLLA scaffold. The percentages of live UCs in the PLLA-UECM scaffold (74.3 ± 2.7%) and the PLLA-SIS scaffold (69.9 ± 3.1%) were significantly higher than that in the PLLA scaffold (37.6 ± 5.5%) (*p* = 0.43; Fig. 2).

### Cell proliferation

UCs were seeded onto PLLA-UECM, PLLA and PLLA-SIS scaffolds at a density of 4 × 10^4^ cells/cm^2^. At day 1, 3, 5, and 7 after seeding, the mitochondrial activity of the cells within the scaffolds was detected by MTT assay. The MTT assay data were transformed to cell number using a standard curve specific to each group. The results suggested that the UCs in each group all maintained a similar growth pattern. However, at day 5 and 7, the number of UCs in the PLLA-UECM scaffold and the PLLA-SIS scaffold showed significant increase compared to that in the PLLA scaffold (Table 2). The number of UCs in the PLLA-UECM and the PLLA-SIS groups were not significantly different at any time point (*p*>0.05).

**Table 2:** Proliferation analysis by the MTT assay. All values are expressed as means ± standard deviation and were analyzed with a one-way ANOVA.

Time (days)	Cell number		
	PLLA-UECM	PLLA	PLLA-SIS
1	6074 ± 2163	5913 ± 1037	5778 ± 1660
3	9120 ± 2710	8213 ± 1600	8970 ± 2915
5	18759 ± 1201*	12996 ± 946	17354 ± 1874*
7	23064 ± 1597*	18146 ± 1030	21667 ± 780*

*p<0.05 versus the PLLA group.

### Phenotype expression of UCs

**Fig 3:**
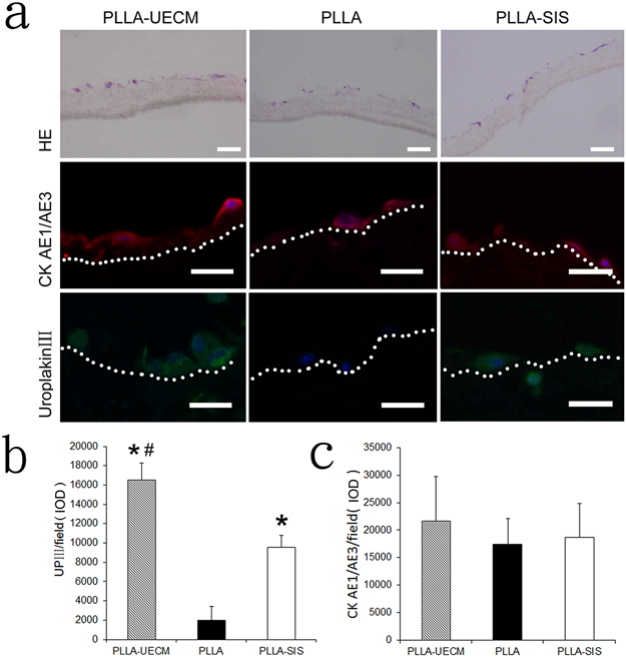
(a) H&E staining showed that the seeded cells remained in the scaffolds and mainly gathered on the surface of the scaffolds. Immunofluorescence staining showed that more cells present in the PLLA-UECM scaffold were positive for UPIII compared to those in the PLLA and PLLA-SIS groups. Image analysis by IPP revealed that there was more UPIII expression in the PLLA-UECM group than in the other two groups (b). However, the expression of the non-specific marker CK AE1/AE3 was not significantly different between groups (c). Inset scale bar = 20 μm. #: *p*<0.05 versus the PLLA-SIS group; *: *p*<0.05 versus the PLLA group. The region below the white dotted line represents the scaffold.

H&E staining showed that the entrapped UCs remained in the three different scaffolds 14 days after seeding. After 14 days of incubation, the seeded UCs mainly formed monolayer on the surface of the scaffolds. In some local areas, a small number of the entrapped cells formed multilayer and infiltrated into the matrix. The UCs mainly gathered close to the surface of the scaffolds. After 14 days of incubation, the phenotypes of the UCs in the three different scaffolds were evaluated by immunofluorescence staining. The UCs in the PLLA-UECM scaffold maintained their phenotype and strongly expressed the UC specific marker UPIII. In contrast, the cells in the PLLA and PLLA-SIS scaffolds expressed less UPIII compared to the cells in the PLLA-UECM scaffold. However, the cells in the three different scaffolds all strongly expressed CK AE1/AE3, which is a non-specific antigen for UCs (Fig. 3). Semi-quantitative analysis by IPP confirmed the phenotype expression of the seeded UCs. Although the bar graph indicates that the expression of CK AE1/AE3 was slightly higher in the PLLA-UECM scaffold compared to the other two controls, the difference between the groups was not significant (*p* = 0.083). However, the expression of UPIII in the PLLA-UECM scaffold was significantly higher than that in the PLLA scaffold or the PLLA-SIS scaffold (*p*<0.05).

**Fig 4:**
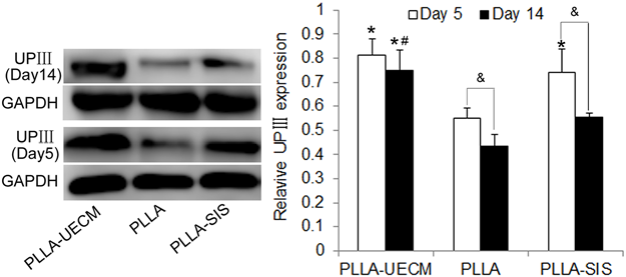
Western blot analysis for the specific marker (UPIII) of UCs. Left: UPIII expression at day 5 and day 14 in the three different scaffolds. Right: Semi-quantitative data for the relative protein expression levels (UPIII /GAPDH). *: *p*<0.05 versus the PLLA group; #: *p*<0.05 versus the PLLA-SIS group. &: *p*<0.05 versus UPIII expression at day 14 in the same scaffold.

To further explore the differences in the expression of UPIII, a specific marker of UCs, western blot analysis was performed at day 5 and 14 post-seeding (Fig. 4). The expression of UPIII was significantly higher in the PLLA-UECM and PLLA-SIS scaffolds than in the PLLA scaffold at day 5. Although the bar graph indicates that the expression of UPIII was higher in the PLLA-UECM scaffold group than in the PLLA-SIS scaffold, the difference was not significant (*p*>0.05). At day 14, the expression of UPIII was significantly higher in the PLLA-UECM scaffold than in the other two groups. In addition, the expression of UPIII in the PLLA scaffold and the PLLA-SIS scaffold was significantly decreased at day 14 when compared to that at day 5. These findings suggest that the expression of UPIII decreased with time when UCs were isolated from their *in vivo* microenvironment. The results also indicate that the PLLA-UECM scaffold can help maintain the specific phenotype of seeded UCs for a longer period of time compared with the other scaffolds.

## Discussion

The ECM is composed of multiple bioactive elements, such as collagen, adhesion molecules, fibronectin and elastin. Some factors, such as VEGF, FGF and TGF-β, in decellularized ECM play important roles in its biological activity [11,20]. Phenotypes and functional characteristics of terminally differentiated cells will be affected when they are isolated from their native tissues and cultured *in vitro* [7,21]. Previous studies have shown that tissue-matched ECM has the ability to promote cell adhesion and proliferation and can maintain cell phenotypes. For example, Zhang YY *et al*. demonstrated that skin, skeletal muscle and liver cells exhibit better proliferation and differentiation in cultures containing ECM from their respective tissues of origin [21]. Each tissue has subtle differences in the ECM structure and composition, which lead to specific cell-ECM interactions. Cell-ECM communication plays an important role in cell differentiation and phenotype maintenance *in vivo*. When cells are isolated from their native tissue and cultured *in vitro*, terminally differentiated somatic cells rapidly lose some of their phenotypic traits. Because of the complex composition of the ECM, it is difficult to completely synthesize in a laboratory using artificial methods.

The biomaterials used to fabricate scaffolds in tissue engineering can be classified into two types: naturally derived materials and synthetic polymers. Naturally derived materials such as collagen and SIS are highly biocompatible and have similar physiological properties to native tissues [22]. However, scaffolds constructed entirely with naturally derived materials often have poor mechanical strength, which ultimately results in collapse after transplantation [23]. Although significant progress has been made in the biomaterials field, current materials do not offer a microenvironment for cells that maintains some of their important characteristics during long-term culture *in vitro* [17,24]. Electro-spinning of PLLA nano-fibers has been adopted to mimic the fibrillar structure of the ECM in urethral tissue engineering [15]. However, because of their hydrophobicity and lack of specific cell-recognizable signals [17], PLLA nano-fibers do not have a favorable surface for cell attachment and differentiation.

In this study, we fabricated a tissue-specific scaffold by blending ureteral ECM with PLLA using an electrostatic spinning method. In addition, the flat electrospun nanofiber meshes could be cut and sew them into cylindrical scaffolds with different diameters and we selected ethylene oxide to sterilize the electrospun scaffolds in this study. There are few suitable methods available to sterilize synthetic high-molecular scaffolds because they are susceptible to morphological degeneration. However, it is crucial for such scaffolds that the meticulous micro- and nano-structures are preserved after sterilization. Previous study found that ethylene oxide has no effect on the initial inherent viscosity and mechanical properties of copolymer [25]. The PLLA-UECM scaffold we fabricated in this study can effectively promoted cell viability and proliferation, and it also maintained the differentiated phenotype of seeded UCs. For example, live/dead staining showed that the absolute number of dead cells in the PLLA group was more than those in the PLLA-UECM or PLLA-SIS group. However, the total number (live + dead) of cells was similar among the groups. The results indicated that the PLLA-UECM scaffold and PLLA-SIS scaffold were more suitable for cell survival than PLLA scaffold. The MTT assay indicated that the cells seeded on the PLLA-UECM scaffold maintained a consecutive growth pattern and significantly increased in number compared to the PLLA scaffold cells. Although these measurements might be biased, it reflected the cell survival and proliferation in a certain extent. WB analysis showed that CK AE1/AE3 (non-specific marker of UCs) and UPIII (specific marker of UCs) expression decreased with time when the UCs were isolated from their native tissue microenvironment. At day 14, the expression of UPIII was significantly higher in the PLLA-UECM scaffold than in the other two scaffolds. In addition, the UPIII expression level was maintained better in the PLLA-UECM scaffold and did not decrease as rapidly as in the other two scaffolds. However, the expression of the non-specific marker CK AE1/AE3 was not significantly different between groups.

The phenomenon that the ureteral tissue-specific scaffold could preserve the differentiated phenotype of UCs when incubated *in vitro* is interesting, but the underlying mechanisms still remain unclear. Multiple bioactive elements in the tissue-specific microenvironment may play an important role in maintaining the specific phenotypes of somatic cells. Therefore, studies that explore the differences between the ECM are needed in the future. Ureteral tissue is composed of two cell types, smooth muscle and UCs. The impact of tissue-specific ECM on the smooth muscle cells is also needed to be explored. In addition, the present research has only studied the impact of flat tissue-specific scaffold on UCs *in vitro*. The effects of cell-cell connection and cell density on the UCs phenomenon were also not detected. In the further studies, we will sew it into a cylindrical scaffold and explored its effects on repairment of injured ureter *in situ*.

## Conclusions

The present study demonstrates that the ureteral tissue-specific scaffold could serve as a potential substrate for the growth of UCs and the maintenance of their differentiated phenotype. The underlying mechanism might be that the PLLA-UECM scaffold providing a substrate that is similar to the *in vivo* microenvironment. In addition, the information from this study also provides a preliminary foundation for tissue engineering-based ureteral reconstruction *in vitro*.
